# First Case of Pyogenic Spondylodiscitis Caused by Gemella sanguinis

**DOI:** 10.7759/cureus.26413

**Published:** 2022-06-29

**Authors:** Kunihiko Hashimoto, Eiji Wada, Kazuma Kitaguchi, Kazuya Ooshima, Kenji Hayashida

**Affiliations:** 1 Department of Orthopedic Surgery, Osaka Police Hospital, Osaka, JPN; 2 Spine and Spinal Cord Center, Osaka Police Hospital, Osaka, JPN

**Keywords:** gemella sanguinis, orthopedic disease, infection, maldi-tof ms, pyogenic spondylodiscitis

## Abstract

A 78-year-old man presented with back pain. Magnetic resonance imaging revealed marrow edema within the L4 and L5 vertebral bodies and a spinal epidural abscess in the spinal canal. The patient was considered to have pyogenic spondylodiscitis at the L4/L5 level. The Gram-positive cocci isolated from blood cultures were subsequently identified as *Gemella* *sanguinis* using matrix-assisted laser desorption ionization-time-of-flight mass spectrometry (MALDI-TOF MS). Symptom improvement was achieved and the infection was eradicated with conservative treatment (treatment with ceftriaxone [CTRX] and minocycline [MINO]). We report the first case of *G. sanguinis*-associated pyogenic spondylodiscitis. MALDI-TOF MS was useful in identifying this uncommon bacterium.

## Introduction

The incidence rate of pyogenic spondylodiscitis, a relatively rare disease, ranges from 0.2 to 2.0 cases per 100,000 people [1]. Surgical site infection during spinal surgery, hematogenous infection, and infection of the surrounding soft tissue most often contribute to the development of pyogenic spondylodiscitis [2]. As reported in past studies, *Staphylococcus aureus* is the most common causative bacterium of pyogenic spondylodiscitis, followed by *Escherichia coli* [2-4]. We herein report a rare case of pyogenic spondylodiscitis caused by *Gemella sanguinis*. Mass spectrometry was effective in identifying this uncommon bacterium.

## Case presentation

**Figure 1:**
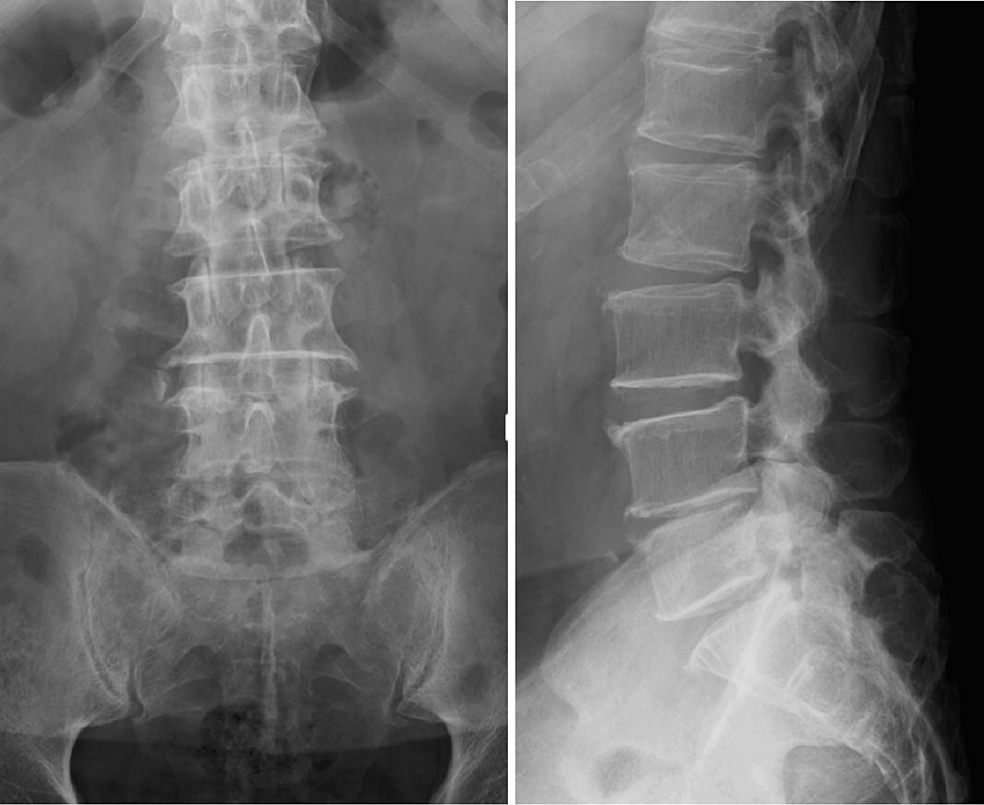
Radiography image of the lumbar spine. The image shows the presence of spondylosis but no signs of radiolucent shadows or fractures.

A 78-year-old man with a history of type 2 diabetes mellitus and chronic hepatitis C presented to our hospital with persistent back pain, which he had been experiencing for two months. He did not have a fever. He had received dental cleanings every month for his dentures. On physical examination, his vital signs were within the normal limits. He had low back pain regardless of his posture and numbness of both lower limbs in the supine position. Laboratory tests revealed a white blood cell count of 1.2 × 104/μL and a C-reactive protein level of 6.01 mg/dL. His HbA1C was 6.7%. His urinalysis results were normal. Radiography of the lumbar spine showed spondylosis but no signs of radiolucent shadows or fractures (Figure 1).

**Figure 2:**
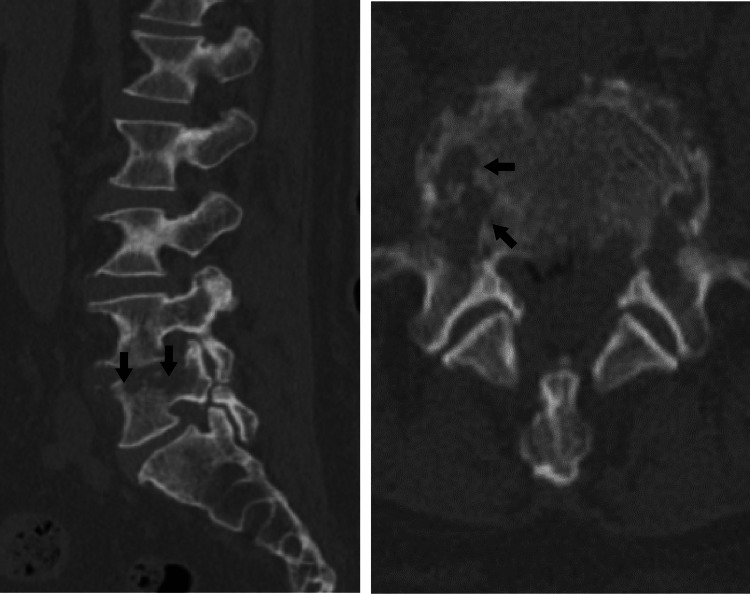
Computed tomography image of the lumbar spine. The image shows destruction of the vertebral body and pedicle (arrows) centered on end plates on the right side of L5.

Computed tomography (CT) revealed destruction of the vertebral body and pedicle centered on the end plates of the fifth lumbar vertebra (L5) (Figure 2).

**Figure 3:**
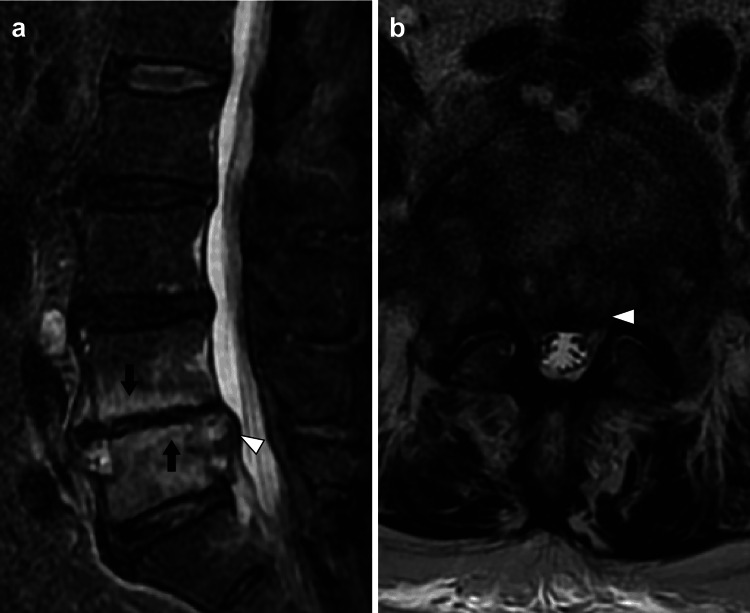
Magnetic resonance image of the lumbar spine. The image shows marrow edema (arrows) within the L4 and L5 vertebral bodies and spinal epidural abscess (white arrowhead) on the short-Ti inversion recovery image (a). Spinal epidural abscess (white arrowhead) in the spinal canal at the level of L5 on the T2-weighted image (b).

Magnetic resonance imaging revealed high signal intensity (marrow edema) in the L4 and L5 vertebral bodies on the short-Ti inversion recovery sequence and a space-occupying lesion (spinal epidural abscess) in the spinal canal at the L5 level (Figure 3).

**Figure 4:**
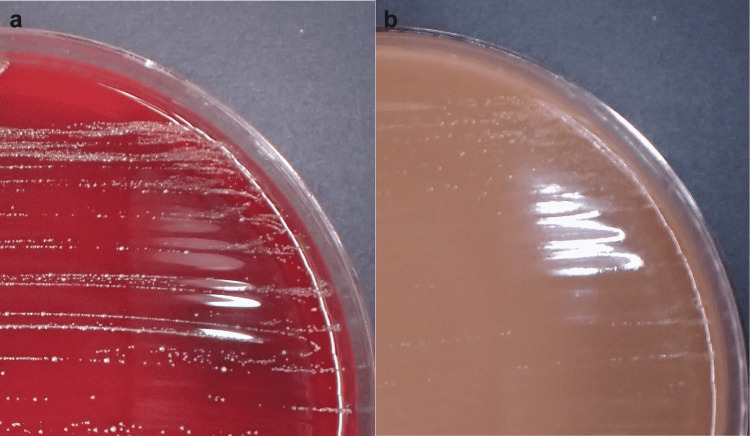
Colonies of Gemella sanguinis on a sheep blood agar plate after incubation for 24 hours at 35 °C (a). Colonies of Gemella sanguinis on a chocolate agar plate after incubation for 24 hours at 37 °C (b).

The patient, in this case, was considered to have pyogenic spondylodiscitis at the L4/L5 level. On the same day, two sets of blood cultures were obtained from different sites. Under fluoroscopy, punctures of the disc were performed from the right side, and a sample was obtained. Assuming it was *Staphylococcus aureus*, treatment with the β-lactam antibiotic cefazolin (CEZ; 2 g/day, intravenously) was started on the first day of hospitalization. The tetracycline antibiotic minocycline (MINO; 200 mg/day, oral), an antibiotic with good bone penetration properties, was also used. He had chronic kidney disease (severe impairment: estimated glomerular filtration rate of 29.6 mL/min/1.73 m^2^); hence, antibiotics were started at low doses. The culture of the incubated disc was negative. Gram staining by the Bartholomew and Mittwer method of the blood indicated Gram-positive cocci. Blood cultures were grown in an atmosphere of 5% CO_2_ on sheep blood agar plates at 35 °C and on chocolate agar plates at 37 °C. After incubation for 24 hours, the growth of small colonies was observed (two sets of blood cultures) (Figure 4).

**Figure 5:**
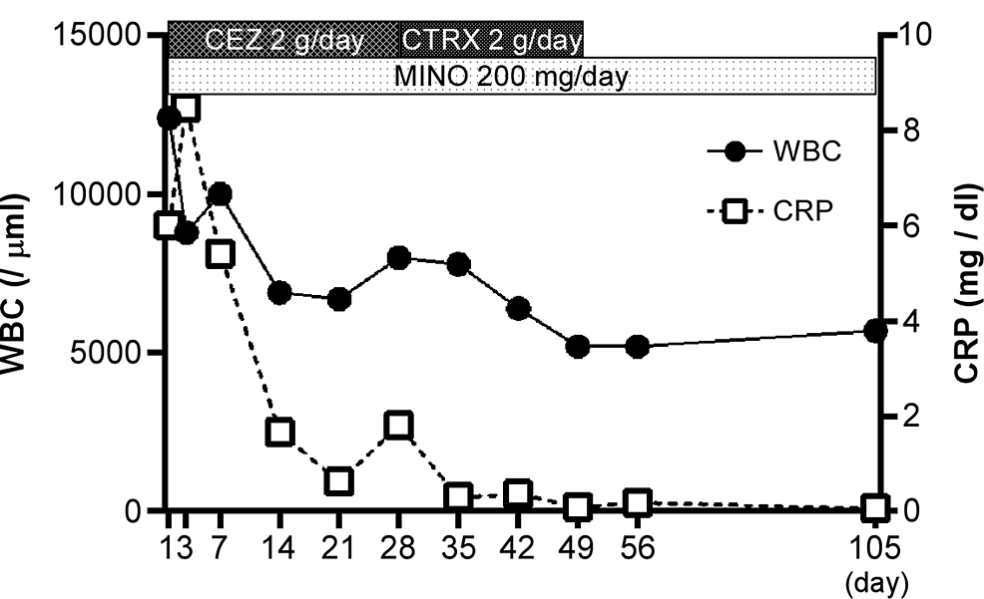
Medical treatment process and laboratory data. WBC: white blood cell count; CRP: C-reactive protein level.

Since the bacterium could not be identified simply by the colony morphology and bacterial structure, matrix-assisted laser desorption ionization-time-of-flight mass spectrometry (MALDI-TOF MS) was used, and these Gram-positive cocci were identified as *G. sanguinis* [5]. A transthoracic echocardiogram revealed no evidence of endocarditis. Due to poor improvement in the inflammatory markers, after approximately four weeks of combined treatment with the two antibiotics, the antibiotic CEZ was switched to ceftriaxone (CTRX) (2 g/day, intravenously). CTRX and MINO were then administered for three weeks. The combined treatment with the two antibiotics was then changed to oral MINO alone (Figure 5).

**Figure 6:**
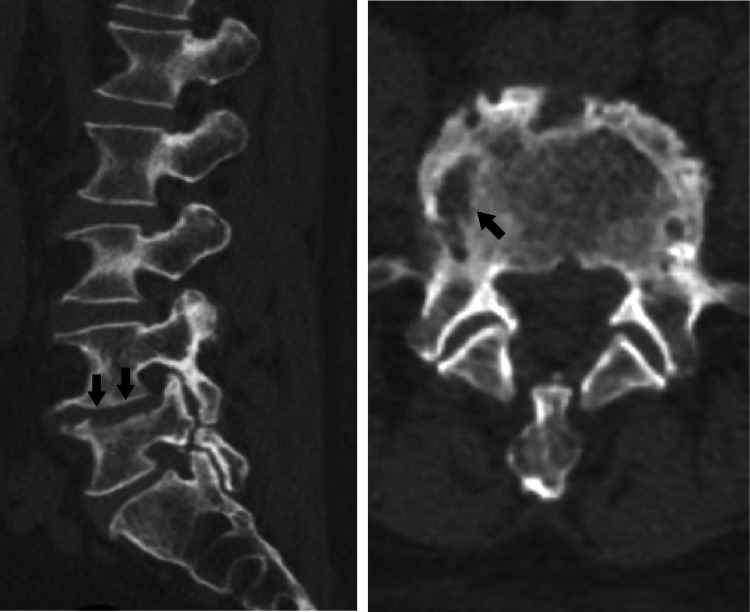
Computed tomography image of the lumbar spine at one-year follow-up. The image shows remodeling of the vertebral body and pedicle (arrows) on the right side of L5.

The patient was discharged from the hospital after eight weeks of treatment. Transthoracic echocardiography performed twice during hospitalization revealed no suspected vegetation in the cardiac valves. At the one-year follow-up, CT findings revealed remodeling of the vertebral body and pedicle in the vertebral body of L5 (Figure 6).

The patient was informed that information about his case would be submitted for publication, and he provided consent.

## Discussion

*G. sanguinis* rarely causes infective endocarditis or prosthetic joint infection, and other infections are even more infrequent [6-10]. There have been several reports of pyogenic spondylodiscitis caused by Gemella species, including *Gemella morbillorum* and *Gemella haemolysans* [11-13]. However, to the best of our knowledge, there are no reports of pyogenic spondylodiscitis caused by *G. sanguinis*.

*G. sanguinis* is a catalase-negative, facultatively anaerobic, Gram-positive, non-spore-forming coccus. Gemella species are found in the normal oral, genitourinary, and gastrointestinal flora. Gemella species include *G. morbillorum*, *G. haemolysans*, *Gemella bergeri*, and *G. sanguinis*, which are differentiated based on biochemical tests and electrophoretic analysis of whole-cell proteins [14]. In our case, colonies were observed to grow after 24 hours of culture in a CO_2_ environment, but species identification could not be performed based on colony morphology alone. The MALDI-TOF MS was used to accurately identify the bacterial species, and the species was identified as *G. sanguinis*. MALDI-TOF MS is one of the most popular mass spectrometry techniques that can be used for the identification of bacteria at the genus, species, and, in some cases, the subspecies level [5]. The database of mass spectrometry is updated regularly, and it is possible to identify rare bacterial species that cannot be identified without gene analysis. Therefore, mass spectrometry is considered extremely useful for bacterial species identification. However, bacterial species not registered in the spectral databases of the MALDI-TOF MS system cannot be identified with this method [5,15]. Another method to identify bacterial species is genetic analysis. Many reports have suggested that infection by *G. sanguinis* stems from an oropharyngeal source, and our patient was a denture wearer and had regular dental cleanings [7,8,10]. It was thought that the background of a compromised host with diabetes mellitus as the underlying disease also contributed to the infection. Even though there have been reports of infective endocarditis [6-9], the transthoracic echocardiogram, in this case, did not confirm vegetation because a transesophageal echo was not done.

In terms of therapy, previous studies of endocarditis or prosthetic joint infection by *G. sanguinis* have reported management by antimicrobial therapy and surgical therapy (valve replacement/debridement and retention) [6-10]. In this case, because the drug susceptibility testing method for the Gemella species has not been established, the susceptibility results tested using MicroFAST 7 and LHB broth (Beckman Coulter) were employed clinically as reference values. The result showed that *G. sanguinis* was a drug-sensitive bacterium (Table 1), and symptom improvement was achieved by the administration of conservative antibacterial treatment. In general, the antimicrobial susceptibility patterns of Gemella species exhibit susceptibility to most β-lactams, vancomycin, and macrolides [10].

**Table 1:** A method for the examination of sensitivity to medications S: susceptible; R: resistant.

Medication	Sensitivity
Benzylpenicillin	S ≤ 0.03
Ampicillin	S ≤ 0.06
Clavulanic acid/Amoxicillin	≤0.25
Cefotiam	≤0.5
Ceftriaxone	S ≤ 0.12
Cefotaxime	S ≤ 0.12
Cefditoren	≤0.06
Cefepime	S ≤ 0.5
Cefozopran	S ≤ 0.12
Meropenem	S ≤ 0.12
Erythromycin	S ≤ 0.12
Azithromycin	S ≤ 0.25
Clindamycin	S ≤ 0.12
Levofloxacin	R > 8
Minocycline	S ≤ 0.5
Sulfamethoxazole-trimethoprim	≤0.5
Vancomycin	S ≤ 0.12

Most patients with pyogenic spondylodiscitis can be managed conservatively. Absolute surgical indications in pyogenic spondylodiscitis include spinal cord or cauda equina compression with the progression of neurological deficits. Relative surgical indications include progressive spinal deformity with biomechanical instability or poor improvement with conservative treatment [1]. The limitation of this study is that the bacteria could not be detected from the puncture site of the disc. The detection rate of percutaneous vertebral biopsy in general infective spondylitis is 77% (range, 47-100%) [16], and so the test was performed in combination with blood cultures. There was sufficient evidence from imaging assessments and blood cultures that the bacterium in question was *G. sanguinis*. This was confirmed by the patient’s improvement after administration of antibiotics that *G. sanguinis* was sensitive to. However, the possibility of blood culture contamination cannot be completely discarded despite the measures taken to reduce this risk, including the use of sterile gloves and masks and the use of 84% ethyl alcohol plus povidone-iodine for skin preparation and blood collection and culture.

## Conclusions

In conclusion, we report a rare case of pyogenic spondylodiscitis caused by *G. sanguinis*. In cases of abnormal conditions in the oral cavity (such as dental diseases), the possibility of *G. sanguinis* should also be considered as a cause of pyogenic spondylodiscitis. Detection techniques such as MALDI-TOF MS are helpful for the identification of such rare bacteria.
